# N-3 PUFAs Protect against Aortic Inflammation and Oxidative Stress in Angiotensin II-Infused Apolipoprotein E^-/-^ Mice

**DOI:** 10.1371/journal.pone.0112816

**Published:** 2014-11-14

**Authors:** Kathryn M. Wales, Kristyn Kavazos, Maria Nataatmadja, Peter R. Brooks, Chloe Williams, Fraser D. Russell

**Affiliations:** 1 Inflammation and Healing Research Cluster, School of Health and Sport Sciences, University of the Sunshine Coast, Maroochydore, Queensland, Australia; 2 Cardiovascular Research Group, Department of Medicine, The Prince Charles Hospital, University of Queensland, Herston, Queensland, Australia; State University of Rio de Janeiro, Biomedical Center, Institute of Biology, Brazil

## Abstract

Abdominal aortic aneurysm is associated with infiltration of inflammatory cells into the aortic wall. The inflammatory response is also evident in animal models, such as apolipoprotein E-deficient (ApoE^-/-^) mice that have been infused with angiotensin II, prior to development of aortic aneurysm. Since omega-3 polyunsaturated fatty acids (n-3 PUFAs) and their metabolites have anti-inflammatory and pro-resolving activity, we hypothesised that dietary supplementation with n-3 PUFAs would protect against inflammatory processes in this mouse model. Twenty C57 and 20 ApoE^-/-^ 3-4 week old male mice were supplemented with a low (0.14%, n = 10/group) or high (0.70%, n = 10/group) n-3 PUFA diet for 8 weeks before 2-day infusion with 0.9% saline or angiotensin II (1000 ng/kg/min). Four ApoE^-/-^ mice on the low n-3 PUFA diet and none of the ApoE^-/-^ mice on the high n-3 PUFA diet showed morphological evidence of abdominal aortic dissection. The plasma concentration of the n-3 PUFA metabolite, resolvin D1 was higher in angiotensin II-infused ApoE^-/-^ mice fed the high, compared to the low n-3 PUFA diet. The number of neutrophils and macrophages infiltrating the abdominal aorta was elevated in ApoE^-/-^ mice on the low n-3 PUFA diet, and this was significantly attenuated in mice that were fed the high n-3 PUFA diet. Most neutrophils and macrophages were associated with dissected aortas. Immunoreactivity of the catalytic subunit of nicotinamide-adenine dinucleotide phosphate (NADPH) oxidase, Nox2, and superoxide were elevated in ApoE^-/-^ mice that were fed the low n-3 PUFA diet, and this was also significantly attenuated in mice that were fed the high n-3 PUFA diet. Together, the findings indicate that supplementation of ApoE^-/-^ mice with a diet high in n-3 PUFA content protected the mice against pro-inflammatory and oxidative stress responses following short-term infusion with angiotensin II.

## Introduction

Abdominal aortic aneurysm (AAA) is a cardiovascular disease that is associated with adventitial inflammation, formation of reactive oxygen species, increased matrix metalloproteinase (MMP) activity, destruction of elastin and collagen, and aortic dilation. Superoxide production is positively correlated with a preoperative risk score for repair surgery, while nicotinamide-adenine dinucleotide phosphate (NADPH) oxidase-stimulated superoxide generation is positively correlated with AAA size [1]. Inflammatory cell infiltration is a common pathophysiological feature of human AAA [2], and is also observed in animal models of AAA, including the angiotensin II-infused, apolipoprotein E-deficient (ApoE^-/-^) mouse [3]. In rodent models of AAA, depletion of neutrophils by the administration of a neutrophil-neutralising antibody decreased the incidence of abdominal aortic dissection [4], the magnitude of aortic dilation [5], and the number of animals developing AAA [5].

Long chain omega-3 polyunsaturated fatty acids (n-3 PUFAs) have anti-inflammatory properties, with health benefits ascribed mainly to eicosapentaenoic acid (EPA) and docosahexaenoic acid (DHA). n-3 PUFAs are primarily obtained from the diet [6], [7], with only low conversion of α-linolenic acid (ALA) to EPA, and EPA to DHA [8]. EPA and DHA are also metabolised by lipoxygenase enzymes to anti-inflammatory and pro-resolving mediators, resolvin E1 and resolvin D1 [9], [10]. These metabolites form in inflammatory exudates and actively initiate a return to normal tissue homeostasis [11]. Infiltration of neutrophils into the peritoneum of mice that were administered with intraperitoneal zymosan A was significantly lower in mice that were pretreated with resolvin D1 compared to mice that were pretreated with vehicle [12].

Infusion of angiotensin II into ApoE^-/-^ mice produces aortic inflammation that can give rise to aortic dissection, and aneurysm in the longer term. Since n-3 PUFAs have anti-inflammatory effects and can be converted to pro-resolving mediators, we hypothesised that supplementation of ApoE^-/-^ mice with a diet containing a high n-3 PUFA composition might lead to 1) an increase in plasma concentration of resolvin D1, 2) a decrease in the infiltration of neutrophils and macrophages into the vessel wall, and 3) a decrease in inflammatory cell-mediated superoxide production. To test this hypothesis, we supplemented C57 and ApoE^-/-^ mice with a diet containing either a low (0.14%), or high (0.70%) n-3 PUFA content for 8 weeks, before infusing the animals for two days with saline or angiotensin II (1000 ng/kg/min) to trigger an inflammatory response. Plasma concentration of resolvin D1, the number of infiltrating neutrophils and macrophages into dissected and non-dissected aortas, the amount of superoxide produced, Nox2 immunoreactivity, and the incidence of abdominal aortic dissection were analysed.

## Materials and Methods

### Dietary supplementation of animals

Twenty C57, BL6 background, and 20 ApoE^-/-^ 3–4 week old male mice were obtained from the Animal Resource Centre, Perth, Australia and transported to the Herston Medical Research Centre, Brisbane. Mice were immediately changed from a cereal grain-based standard rodent diet containing 0.02% EPA, 0.05% DHA, and total n-3 and n-6 PUFA content of 0.37% and 1.31% (Specialty Feeds, WA, Australia), to a cereal grain-based diet containing either a low or high n-3 PUFA content (Table 1). Feed was autoclaved, and analysed for fatty acid content using gas chromatography-mass spectrometry (GC-MS). Briefly, 0.5 g of crushed feed pellets was combined with 4.5 mL of hexane containing 0.2 mg 2,6-di-tert-butyl-4-methylphenol (BHT). Samples were stored in the dark, overnight at 4°C with occasional shaking. Supernatant (0.1 mL) was combined with 1 mL of 1 mol/L lithium methoxide, and incubated at 23°C for 1 h with occasional shaking. Hexane (1 mL) was added, and the methanol layer allowed to partition. This was removed, and 0.5 mL milliQ water was added. The hexane extract was withdrawn for analysis. GC-MS analysis was carried out using a PerkinElmer Clarus 580 gas chromatograph coupled to a PerkinElmer Clarus SQ 8S mass spectrometer. The column was an Elite 5-MS 30 m×0.25 mm×0.25 µm, carrier gas was helium at 1.0 mL/min with a 30∶1 split ratio. The injector was at 300°C, with a temperature program of 120°C initial, held for 0.5 min, ramping at 10°C/minute until 310°C, and then holding for 2.0 min. The mass spectrometer scanned over 40 to 350 m/z+ for 5.0 to 21.5 min.

**Table 1:** Key nutritional content of low and high n-3 polyunsaturated fatty acid (n-3 PUFA) diets used with the C57 and apolipoprotein E^-/-^ mice.

Nutritional parameter	Low n-3 PUFA diet	High n-3 PUFA diet
Protein	20%	20%
Total fat	4.70%	4.70%
Crude Fibre	4.90%	4.90%
Digestible energy	14.4 MJ/kg	14.4 MJ/kg
α-Linolenic acid	0.13%	0.28%
Eicosapentaenoic acid	negligible	0.07%
Docosahexaenoic acid	negligible	0.30%
Arachidonic acid	0.03%	0.02%
Total n-3 PUFA	0.14%	0.70%
Total n-6 PUFA	1.23%	1.24%

Data from manufacturer's calculated nutritional data sheet (Specialty Feeds, WA, Australia).

### Infusion of saline or angiotensin II into mice

Mice were fed a low or high n-3 PUFA diet for 8 weeks, and then infused with saline (C57) or angiotensin II (ApoE^-/-^; 1000 ng/kg/min) for two days. This study was carried out in strict accordance with the recommendations in the Guide for the Care and Use of Laboratory Animals of the National Institutes of Health. The protocol was approved by the Animal Ethics Committee of the University of the Sunshine Coast (Permit Number: AN/A/13/70), and ratified by the University of Queensland Animal Ethics Committee (Permit Number: USC/008/13/USC). All surgery was performed under sodium pentobarbital anesthesia, and all efforts were made to minimize suffering.

Mice were anaesthetised by intraperitoneal injection of sodium pentobarbital (32.5 µg/g body weight in 0.9% saline). The interscapular region of the mouse was shaved using a scalpel, and swabbed using 70% ethanol which was allowed to dry. An incision (∼7 mm in length) was made in the skin using scissors, and a small, subcutaneous pocket was opened by blunt dissection. An osmotic minipump (Alzet, model 1002; CA, USA), primed to deliver either saline (0.9%) or angiotensin II (1000 ng/kg/min), was inserted into the opening. The incision was closed using silk 3/0 sutures, with animals receiving immediate intraperitoneal analgesic (buprenorphine, 330 ng/g body weight). All animals exhibited normal mobility and feeding behaviour post-surgery. Animals were maintained on the same low or high n-3 PUFA diet for two days. At the conclusion of this time, animals were anaesthetised with pentobarbital (325 µg/g body weight in 0.9% saline), and the heart was exposed for blood collection.

### Determination of plasma cholesterol concentration

Duplicate (5 µL) samples of MilliQ water (blank), plasma, Data-Cal™ chemistry calibrator (4.97 mM), Data-Trol N™ normal control serum (mean 4.05 mM; expected range 3.65–4.46 mM) and Data-Trol A™ abnormal control serum (mean 6.63 mM; expected range 5.97–7.29 mM) were combined with Infinity™ cholesterol liquid stable reagent (500 µL) and incubated for 20 min at 22°C. Aliquots (200 µL) were transferred to a 96-well microplate and absorbance was measured at 490 nm. The mean blank absorbance was subtracted from the mean of each duplicate sample. Total serum cholesterol was calculated using the equation below. The intra-assay variation was 5.22% (*n* = 30). 
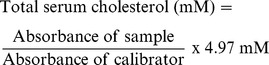



### Determination of plasma triglyceride concentration

Duplicate (5 µL) samples of MilliQ water (blank), plasma, Data-Cal™ chemistry calibrator (1.21 mM), Data-Trol N™ normal control serum (mean 1.15 mM; expected range 0.95–1.35 mM) and Data-Trol A™ abnormal control serum (mean 1.68 mM; expected range 1.48–1.88 mM) were combined with triglyceride reagent (200 µL, 37°C). Aliquots (160 µL) were transferred to a 96-well microplate, and this was incubated at 37°C for 20 min. Absorbance was measured at 490 nm. The mean blank absorbance was subtracted from the mean of each duplicate sample. Triglyceride concentration was calculated using the equation below. The intra-assay variation was 8.03% (*n* = 30). 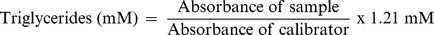



### Determination of docosahexaenoic and eicosapentaenoic acids in erythrocyte membrane phospholipids

Whole blood was collected by cardiac puncture by insertion of a 27 G needle and syringe into the apex of the heart. The blood was decanted into 2 ml EDTA vacutainer tubes, and centrifuged at 1500×g for 10 min at 22°C. Erythrocytes and plasma were placed in separate eppendorf tubes, frozen on dry ice, and stored in a −80°C freezer until use. Blood was not collected from one ApoE^-/-^ mouse on the low n-3 PUFA diet that died of a ruptured abdominal aorta, and one ApoE^-/-^ mouse on the high n-3 PUFA diet that died from a ruptured thoracic aorta.

GC-MS was used to determine the ratio of DHA or EPA to all other fatty acids in erythrocyte membrane phospholipids. Briefly, erythrocytes (∼300 µL) were placed in a glass tube with 600 µL methanol containing 0.02% BHT, and membranes were disrupted by mechanical homogenisation using a glass rod for 1 min. Samples were covered with nitrogen, sealed and placed on ice for 30 min. Chloroform (600 µL) was added, cells were homogenised as before, and samples were stored on ice with nitrogen for 30 min. Tubes were centrifuged at 3000×g for 5 min at 4°C. The homogenisation procedure was repeated twice, but using 300 µL volumes of methanol/BHT and chloroform, and 10 min incubations of tubes on ice. The lipid supernatant was collected after each centrifugation step, and placed on ice. The pooled lipid layer (1 mL) was combined with 800 µL chloroform and 460 µL of 0.05 M KCl, vortexed in the presence of nitrogen, and centrifuged at 3000×g for 10 min at 4°C. The bottom lipid layer was transferred to a 2 mL GC-MS vial and dried under a stream of nitrogen. The sample was hydrolysed with a 500 µL solution containing 9 M HCl, milliQ water, and acetonitrile in a 1∶1∶18 ratio, and 0.05% BHT. Samples were covered with nitrogen, sealed, and incubated overnight at 65°C. The sample was dried under a stream of nitrogen, freeze dried for 20 min, and then combined with hexane (250 µL) containing derivatising agent, N-(tert-butyldimethylsilyl)-N-methyltrifluoroacetamide, with 1% TBDMSCI, 97% (15 µL). Samples were covered with nitrogen, incubated for 2 h at 37°C, and then transferred to 2 mL GC-MS vials. GC-MS was carried out as described above, with the following modification: the split ratio was 10∶1 on injection, opening to 30∶1 after 1.0 min. The injector was at 300°C with a temperature program of 170°C initial, ramping at 6.0°C/min until 310°C, and then holding for 5.0 min. The mass spectrometer scanned over 45 to 450 m/z+ for 4.0–28.2 min.

### Determination of plasma resolvin D1 concentration

Resolvin D1 was measured in plasma samples using a resolvin D1 enzyme immunoassay kit (Cayman Chemical), with comparison against resolvin D1 standards (3.3–2000 pg/mL). Data was compared across treatment groups. ApoE^-/-^ mice that were fed the low n-3 PUFA diet were further analysed for resolvin D1 levels, with segregation according to whether the aorta was dissected or non-dissected, as determined by histological examination.

### Tissue morphology

The abdominal aorta was harvested from the mice, and dissected into suprarenal and infrarenal segments. Tissues were fixed in 10% formalin for 48 h before dehydration and embedding in paraffin. Tissue sections (4 µm) were cut using a microtome, and collected on gelatin-coated microscope slides. Sections were deparaffinised by consecutive changes in xylene (10 min, 2×2 min) and absolute ethanol (3×2 min).

### Quantitation of infiltrating neutrophils and macrophages using immunohistochemistry

Antigenic sites were unmasked by incubation of deparaffinised tissue sections in sodium citrate buffer (tri-sodium citrate, 11.4 mM, containing 0.05% tween-20; pH 6.0) at 95–100°C for 20 min. Sections were cooled in the sodium citrate buffer to ∼45°C before rinsing with distilled water and incubating with 0.3% H_2_O_2_ for 30 min at 23°C. Sections were washed in phosphate buffer solution (PBS; NaCl, 137, KCl, 2.7, Na_2_HPO_4_, 10.1, KH_2_PO_4_, 1.8 mM, Tween-20, 0.05%; pH 7.5; for neutrophil staining) or Tris buffer saline (TBS; for macrophage staining) for 5 min at 23°C, blocked with 1.5% rabbit serum for 20 min at 23°C, and then exposed to rat anti-mouse neutrophil antibody (NIMP-R14; Abcam, Cambridge, UK; 2 µg/mL in PBS containing 0.1% BSA), rat anti-mouse mac-3 antibody (clone m3/84, BD Pharmingen, USA; 0.625 µg/mL in TBS), or antibody diluting buffer alone, for 30 min at 23°C. Neutrophil staining was revealed using a Vectastain Elite ABC kit, and SIGMA*FAST* DAB with Metal Enhancer. Macrophage staining was revealed using 3-amino-9-ethylcarbazole in N,N-dimethyl formamide. Sections were mounted with coverslips, and viewed using a Nikon Eclipse Ti microscope. Each aorta was photographed at four separate regions (for neutrophils) or whole vessel (for macrophages) using a 20× objective lens. The number of neutrophils and macrophages present in the adventitia was determined, and expressed as number/mm length of medial-adventitial surface.

### Vascular production of superoxide and expression of Nox2

Tissue sections were stained for superoxide using 5 µM dihydroethidium (DHE; 20 min, 37°C), washed in Dulbecco's phosphate buffer solution (3×5 min, 23°C) and mounted with coverslips. Slides were viewed using a Nikon Eclipse Ti microscope (ex/em 520/610 nm), and photographed using a Nikon DS-Fi2 camera attachment. An 8-image z stack was compiled from the 4 µm tissue sections. Each vessel was photographed at four separate locations using a 20× objective lens, and quantitated for DHE fluorescence intensity using Image J 1.44p software (NIH, USA).

Nox2 expression was examined using immunohistochemistry. Tissue sections were blocked with 1.5% goat serum in PBS (20 min, 23°C) before incubating with an antibody directed against Nox2/pg91^phox^ (Abcam, Cambridge, UK; 2 µg/mL in PBS containing 0.1% BSA), or with antibody diluting buffer only, for 30 mins at 23°C. Nox2 expression was revealed using the Vectastain Elite ABC kit, and SIGMA*FAST* DAB with Metal Enhancer. Each vessel was photographed at two separate locations using a 20× objective lens. Stain intensity was quantitated using Image J software.

### Mortality from aortic rupture

A second cohort of animals was fed a low (n = 27) or high n-3 PUFA diet (n = 26) for 8 weeks and then infused with angiotensin II (1000 ng/kg/min) for 2 weeks. Mortality rate was assessed. Post-mortem examination was used to determine whether the animal died from rupture of the thoracic or abdominal aorta.

### Statistical analysis

Data was analysed using one-way ANOVA Contrasts, and one-way ANOVA with Tukey post-hoc test (IBM SPSS Statistics, version 21). Comparison of the incidence of aortic dissection was analysed using Fisher exact probability test. Data is presented as mean±SEM.

## Results

### n-3 PUFA content of the rodent feed, mass of feed consumed, and mouse body weight

**Figure 1:**
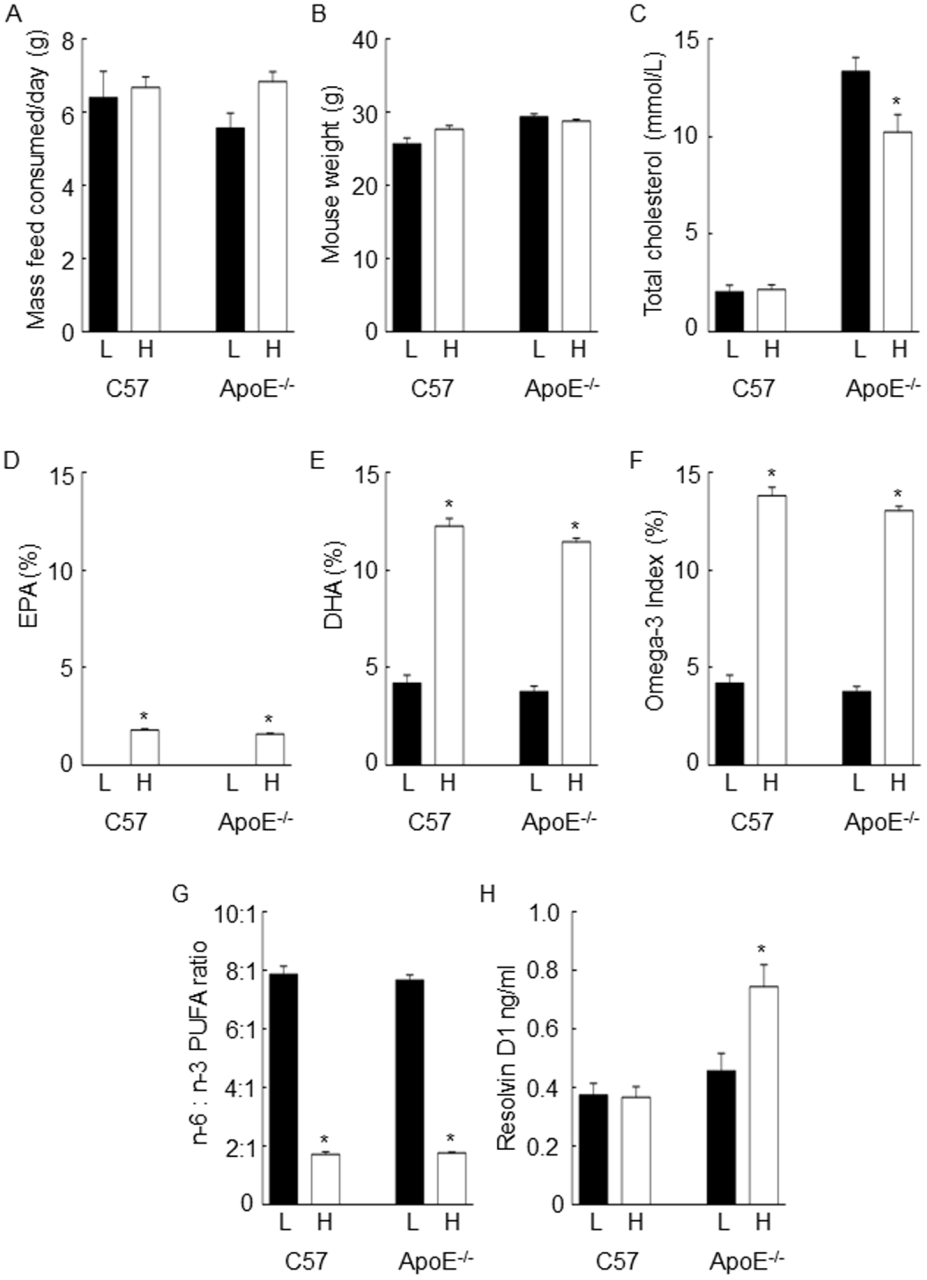
Effect of dietary omega-3 polyunsaturated fatty acids (n-3 PUFAs) on mouse feeding behaviour, fatty acid incorporation in membrane phospholipids, and plasma resolvin D1 concentration. Mice were fed for 8 weeks with a low or high n-3 PUFA diet, and infused for 2 days with either saline (C57 mice) or angiotensin II (ApoE^-/-^ mice). Amount of feed consumed (A), and mouse weight after 8-week dietary supplementation (B) were unaffected by diet. Total cholesterol was elevated in ApoE^-/-^ mice, and this was attenuated by the high n-3 PUFA diet (C). The percentage of total fatty acids containing DHA (D), EPA (E), and DHA plus EPA (n-3 index; F) was significantly greater in mice receiving the high, compared to the low n-3 PUFA diet. The ratio of n-6 to n-3 PUFAs was lower in the animals receiving the high, compared to the low n-3 PUFA diet (G). The plasma concentration of resolvin D1 was higher in ApoE^-/-^ mice receiving a high, compared to the low n-3 PUFA diet, with no difference in C57 mice (H). *, P<0.05. L, low n-3 PUFA diet; H, high n-3 PUFA diet.

Calculated data obtained from the manufacturer of the low n-3 PUFA diet showed ALA to be the main source of n-3 PUFAs, with EPA and DHA levels below the limit of detection (Table 1). The high n-3 PUFA diet contained more DHA than EPA. The mass of feed consumed, and mouse bodyweight for C57 and ApoE^-/-^ mice was the same for animals that were fed the low and high n-3 PUFA diets (Figure 1A, B).

### Effect of n-3 PUFA diet on plasma cholesterol and triglyceride levels

Total plasma cholesterol was greater in ApoE^-/-^ angiotensin II-infused mice, compared to C57 saline-infused mice, across both dietary groups, and lower in ApoE^-/-^ angiotensin II-infused mice fed the high, compared to the low n-3 PUFA diet (P<0.05; Figure 1C). There was no significant difference in plasma triglyceride concentration between ApoE^-/-^ angiotensin II-infused mice and C57 saline-infused mice, or between ApoE^-/-^ angiotensin II-infused mice fed the high or low n-3 PUFA diets (Table S1).

### Effect of n-3 PUFA diet on membrane phospholipid n-3 PUFA content and plasma resolvin D1 concentration

The incorporation of EPA and DHA into cell membrane phospholipids was confirmed by GC-MS. Consistent with the higher dietary intake of DHA than EPA, a higher proportion of DHA than EPA was incorporated into erythrocyte membrane phospholipids. In animals receiving the low n-3 PUFA diet, EPA was below the limit of detection in the membrane phospholipids whereas DHA comprised about 4% of total membrane phospholipid fatty acids (Figure 1D, E). The n-3 index was higher (P<0.05; Figure 1F), and n-6:n-3 PUFA ratio was lower (P<0.05; Figure 1G) in mice that received the high, compared to the low n-3 PUFA diet, for C57 and ApoE^-/-^ groups. We hypothesised that supplementation of mice with the high n-3 PUFA diet would result in a greater production of resolvin D1 than mice that were supplemented with the low n-3 PUFA diet. This finding was observed in mice that were exposed to the pro-inflammatory stimulus (ApoE^-/-^ angiotensin II-infused mice, P<0.05; Figure 1H). There was no difference in the plasma concentration of resolvin D1 for saline-infused C57 mice receiving the two different diets (Figure 1H).

### Effect of n-3 PUFA diet on inflammatory cell infiltration and aortic dissection

**Figure 2:**
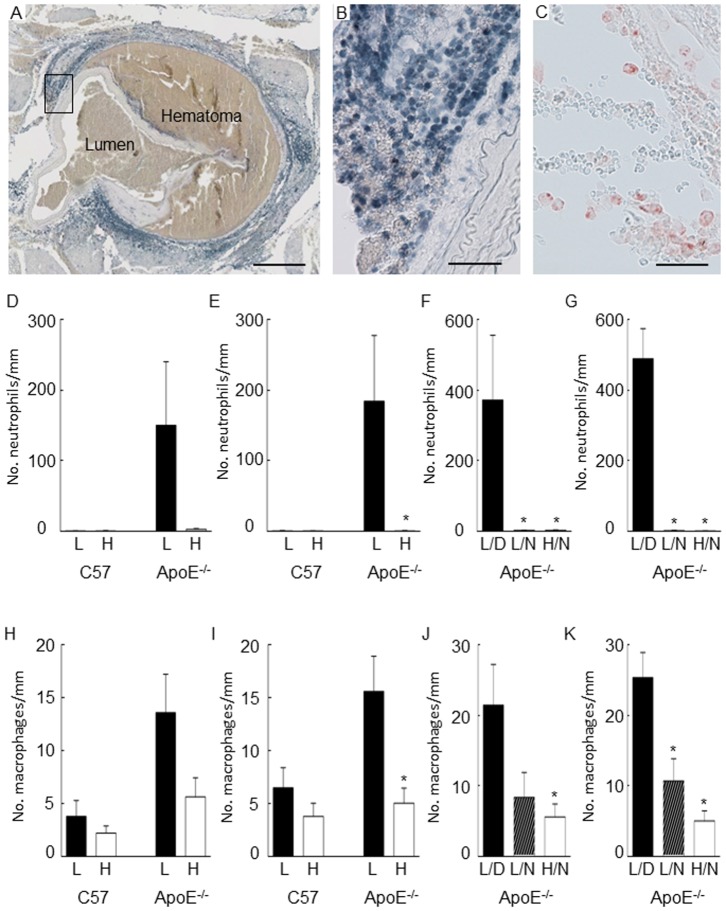
Effect of dietary omega-3 polyunsaturated fatty acids (n-3 PUFAs) on inflammatory cell infiltration of the abdominal aorta. Some of the ApoE^-/-^ mice fed the low n-3 PUFA diet had a dissected aorta, with morphological evidence of a large hematoma (A). Immunoreactive staining for neutrophils (B) and macrophages (C) was detected in the adventitial of the aorta. Neutrophil infiltration was prominent in ApoE^-/-^ mice that were fed the low n-3 PUFA diet (D, suprarenal; E, infrarenal region), and this was mainly associated with dissected aortas (F, suprarenal; G, infrarenal region). Macrophage infiltration was also prominent in ApoE^-/-^ mice that were fed the low n-3 PUFA diet (H, suprarenal; I, infrarenal region), and this was also mainly associated with dissected aortas (J, suprarenal; K, infrarenal region). Scale bar, 400 µm (A), 50 µm (B, C). L, low n-3 PUFA diet; H, high n-3 PUFA diet; L/D, low n-3 PUFA diet in dissected aortas; L/N, low n-3 PUFA diet in non-dissected aortas; H/N, high n-3 PUFA diet in non-dissected aortas.

Some abdominal aortas dissected, as evidenced by the formation of a large intramural hematoma at the medial-adventitial border (Figure 2A). Four of the ten ApoE^-/-^ mice infused with angiotensin II and fed the low n-3 PUFA diet showed morphological features of abdominal aortic dissection. In contrast, none of the ten ApoE^-/-^ mice infused with angiotensin II had a dissected abdominal aorta. Neutrophils (Figure 2B) and macrophages (Figure 2C) were identified within the adventitial region of the aorta in mice with abdominal aortic dissection, with a greater number of neutrophils than macrophages detected in the infrarenal aorta of mice fed the low diet (P<0.05; Figure 2G, K). We examined whether the high n-3 PUFA diet and/or the corresponding increase in plasma resolvin D1 might be associated with reduced infiltration of inflammatory cells in the adventitial region of the aorta. Fewer infiltrating neutrophils and macrophages were observed in the infrarenal aorta of ApoE^-/-^ mice that received the high, compared to the low n-3 PUFA diet (P<0.05; Figure 2E, I). Most neutrophils and macrophages were detected in dissected suprarenal and infrarenal aortas, with neutrophils rarely detected in non-dissected vessels (Figure 2F, G, J, K).

### Effect of n-3 PUFA diet on markers of oxidative stress

**Figure 3:**
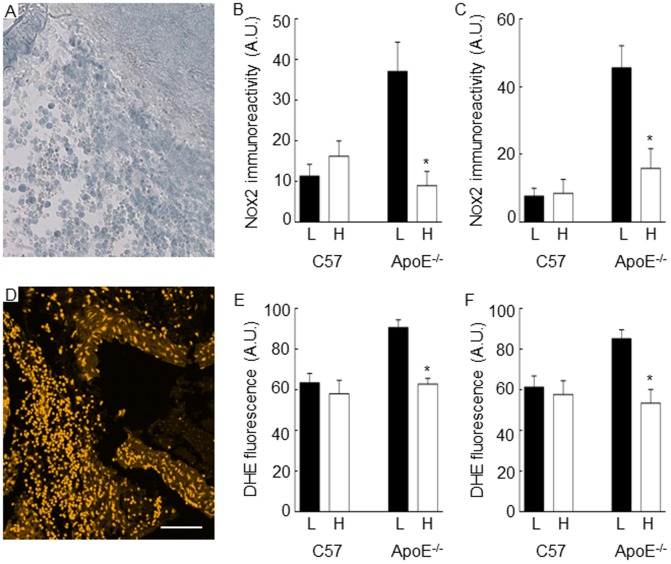
Quantitation of Nox2-immunoreactivity and superoxide production in inflammatory cells infiltrating the abdominal aorta of C57 saline-infused, and apolipoprotein E-deficient (ApoE^-/-^) angiotensin II-infused mice. Mice were fed a low or high omega-3 polyunsaturated fatty acid (n-3 PUFA) diet for 8 weeks. NOX2 immunoreactive staining was evident in infiltrating inflammatory cells of ApoE^-/-^ angiotensin II-infused mice on the low diet (A). Expression levels of Nox2 were lower in the suprarenal (B) and infrarenal aorta (C) of ApoE^-/-^ angiotensin II-infused mice that received the high, compared to the low n-3 PUFA diet. Intense dihydroethidium (DHE) fluorescence, a marker for superoxide, was detected in adventitial neutrophils of ApoE^-/-^ angiotensin II-infused mice on the low diet (D). DHE fluorescence intensity was lower in the suprarenal (E) and infrarenal aorta (F) of ApoE^-/-^ angiotensin II-infused mice that received the high, compared to the low n-3 PUFA diet. * P<0.05. L, low n-3 PUFA diet; H, high n-3 PUFA diet; A.U., Arbitrary units.

Nox2 immunoreactivity was observed in adventitial inflammatory cells of abdominal aortas (Figure 3A). The intensity of immunoreactive Nox2 staining was elevated in these cells from angiotensin II-infused ApoE^-/-^ mice that were fed the low n-3 PUFA diet (Figure 3B, C). Nox2 immunoreactivity was lower in mice fed the high, compared to low n-3 PUFA diet for both suprarenal and infrarenal regions of the abdominal aorta (P<0.05; Figure 3B, C). Consistent with the expression of Nox2 in inflammatory cells of the adventitia, these cells also had a high level of superoxide production per cell, as indicated by intense fluorescence following incubation of sections with dihydroethidium (Figure 3D). Consistent also with the ability of the high n-3 PUFA diet to attenuate Nox2 expression, this diet also lead to a lower production of superoxide per inflammatory cell in ApoE^-/-^ angiotensin II-infused mice, compared to animals that were fed the low n-3 PUFA diet for both suprarenal and infrarenal aortic segments (P<0.05; Figure 3E, F). The amount of superoxide that was detected in the ApoE^-/-^ angiotensin II-infused mice that were fed the high n-3 PUFA diet was not different to the levels detected in the C57, saline-infused control mice (Figure 3E, F).

### Effect of n-3 PUFA diet on incidence of aortic rupture

**Figure 4:**
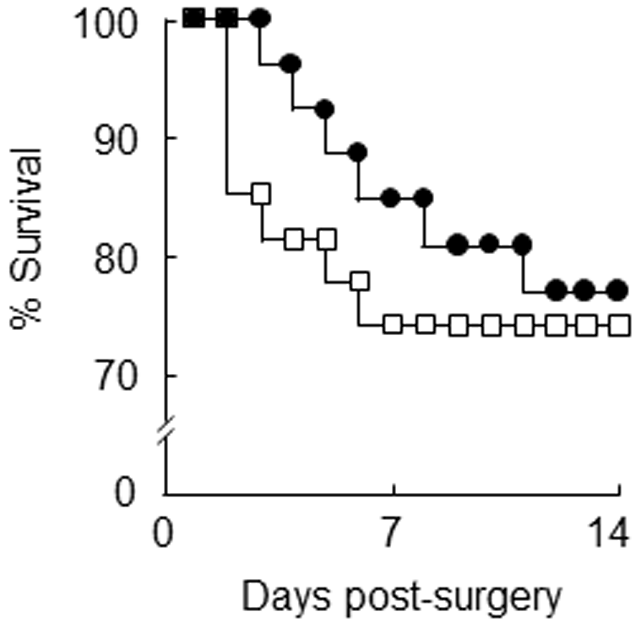
Survival curves showing total mortality associated with ruptured thoracic or abdominal aorta in mice that were fed a diet containing a low or high omega-3 polyunsaturated fatty acid (n-3 PUFA) content for 8 weeks prior to infusion of angiotensin II (1000 ng/kg/min) for 2 weeks. The high n-3 PUFA diet (n = 26, filled circles) caused a trend for delay to time of rupture compared to low n-3 PUFA diet (n = 27, open squares).

To determine whether there was a survival benefit of the high n-3 PUFA diet, a second cohort of ApoE^-/-^ mice were fed the low (n = 27) or high n-3 PUFA diet (n = 26) for 8 weeks and then infused with angiotensin II for 2 weeks, whilst maintaining the same diets. Seven mice that were fed the low n-3 PUFA diet died after rupture of the abdominal aorta, with no deaths attributed to ruptured thoracic aorta. Three mice fed the high n-3 PUFA diet died after rupture of the abdominal aorta, and three mice died after rupture of the thoracic aorta. Although there was a trend for fewer deaths attributed to ruptured abdominal aorta, this was not significant (two tailed Fishers Exact Probability test, P>0.05). Survival curves for total mortality attributed to ruptured abdominal and thoracic aorta in mice that were fed the high n-3 PUFA diet showed a trend for delay to time of rupture compared to mice that were fed the low n-3 PUFA diet (Figure 4).

## Discussion

Omega-3 fatty acids have anti-inflammatory effects, and can be metabolised to pro-resolving mediators. The aim of this study was to determine whether supplementation of ApoE^-/-^ mice with a high n-3 PUFA diet might protect mice against vascular inflammation and oxidative stress. In this study, ApoE^-/-^ mice were administered a diet that contained either a low or high n-3 PUFA content for 8 weeks. This duration of treatment was sufficient to increase the proportion of total fatty acids containing both EPA and DHA in erythrocyte membrane phospholipids (Figure 1) [13]. The high n-3 PUFA diet was formulated to contain more DHA than EPA, as cardiovascular health benefits have been attributed mainly to this fatty acid [14], and DHA is regarded to be the most important functional n-3 PUFA [8]. The n-3 index and n-6:n-3 PUFA ratio for mice fed the low n-3 PUFA diet was similar to that reported for Western populations in which there is a sub-optimal dietary intake of n-3 PUFAs (n-3 index, 3.2-3.84%; n-6:n-3 PUFA ratio, 9.6∶1; [15], [16]), while the high n-3 PUFA diet provided a lipid profile that was similar to a Japanese population in which 65% of men and women aged 50-60 years consume ≥3 fish meals per week (n-3 index, 10.6%; n-6:n-3 PUFA ratio, 3.75∶1 [17]).

The low n-3 PUFA feed contained a negligible amount of DHA, as determined by manufacturer's calculated composition, and confirmed by our own GC-MS data. Despite this, GC-MS analysis of the erythrocyte membrane phospholipid fatty acid composition revealed that DHA comprised ∼4% of total membrane fatty acids. The main n-3 PUFA in the feed was ALA, raising the possibility that some of the DHA may have been derived from enzymatic conversion of ALA. Although *in vivo* conversion of ALA to DHA is normally inefficient (<0.1% in humans) [8], conversion efficiency appears to be elevated when a diet that is deficient in DHA is consumed [8], [18]. A second possible source of DHA is the standard feed that was consumed by mice prior to their randomisation to the low and high n-3 PUFA diets. However, it is unlikely that residual DHA will be present in membrane phospholipids 8 weeks after removal of the standard diet, as membrane phospholipid DHA-content declines rapidly after removal (ie. washout) of a diet that is fortified with n-3 PUFAs [13]. Importantly for this study, incorporation of EPA and DHA into membrane phospholipids was significantly greater in mice receiving the high, compared to low n-3 PUFA diets.

Since resolvin D1 is produced from DHA during an acute inflammatory challenge [19], [20], we predicted that the combination of ApoE^-/-^ genetic background with infusion of angiotensin II would increase the plasma concentration of resolvin D1 if the mice were supplemented with the high n-3 PUFA diet. Our findings supported this hypothesis. In control mice that did not receive a stimulus for inflammation, plasma resolvin D1 levels remained low for mice on both diets. These findings indicate that dietary supplementation with DHA alone is not sufficient to drive resolvin D1 production, and that a combination of dietary DHA supplementation plus pathological stimulus is needed. The requirement for an inflammatory stimulus to produce pro-resolving mediators has been reported previously for the lipoxins [21]. In that study, lipoxins were produced by activated neutrophils from patients with inflammatory disease, but not from healthy subjects. In a mouse model of ischaemic acute kidney injury, plasma resolvin D1 concentration was elevated in mice with kidney injury only if the animals were also administered exogenous DHA [22]. The dual effect of deleting the ApoE gene, and infusing mice with angiotensin II, provides the required pro-inflammatory stimulus to increase plasma resolvin D1 levels in the presence of a high dietary intake of n-3 PUFAs.

Angiotensin II contributes to the pathology observed in the aorta by activating NADPH oxidase, and by upregulating NOX isoforms and their accessory proteins to increase production of reactive oxygen species [23]. Hypercholesterolemia caused by ApoE gene deletion leads to upregulation of AT_1_ receptors [24], which would be expected to facilitate inflammation and oxidative stress through the activation of matrix metalloproteinases and production of reactive oxygen species. In the inflammatory milieu, the n-3 PUFA DHA is converted to resolvin D1 [25]. In the biosynthetic pathway, DHA is oxidised in the presence of 15-lipoxygenase and peroxidase to 17S-hydroxy-4Z,7Z,10Z, 13Z,15E, 19Z-docosahexaenoic acid (17S-HDHA), a precursor of the D-series resolvins [26]. The identification of increased circulating concentration of resolvin D1 may therefore be indicative of an activated pathway for the family of D-series resolvins, and thus likely to signify the production of additional resolvin molecules in the ApoE^-/-^ mouse model.

In ApoE^-/-^, angiotensin II-infused mice, abdominal aortic dissection was found to precede the formation of abdominal aortic aneurysms [3]. In that study, early pathological changes occurred between days 1–4 post-infusion with 1000 ng/kg/min angiotensin II, and included medial and adventitial infiltration of macrophages. Neutrophil infiltration was not detected [3], in contrast to our study which showed evidence for the presence of neutrophils in the adventitial region. Aortic dissection and hematoma formation were observed in both studies, although at an earlier time-point in this study (2 days compared to 4–10 days) [3].

Infiltrating inflammatory cells may contribute to the development of abdominal aortic dissection. Macrophages release MMP-9 which degrades collagen [27], while neutrophils produce superoxide, H_2_O_2_, and MMP-9, with diminished expression of catalase [2], [4], [28]. In rodent models of acute peritonitis, n-3 PUFAs and resolvin D1 inhibited the stimulated migration of neutrophils into the peritoneal cavity [12], [29]–[31]. Resolvin D1 also stimulates phagocytic function in macrophages; an effect that is mediated by the activation of high affinity formyl peptide receptor 2 (FPR2/ALX) and GPR32 receptors [10]. The pro-resolving activity of resolvin D1 involves FPR2/ALX and GPR32 receptor-dependent regulation of microRNAs, to modulate expression of a number of cytokines and chemokines [29], [30]. We were therefore interested to see whether supplementation of mice with a high n-3 PUFA diet might attenuate the infiltration of neutrophils into the aortic wall after angiotensin II infusion. The findings showed that abdominal aortic dissection was only observed in angiotensin II-infused mice that received the low n-3 PUFA diet (4/10 mice), with no evidence for this in angiotensin II-infused mice receiving the high n-3 PUFA diet (P = 0.087). Neutrophil infiltration was predominantly associated with dissected aortas; a finding consistent with the greater neutrophil infiltration of human dissected aortas (Stanford type A and type B) compared to non-dissected aortas [4].

Activated neutrophils are a source of reactive oxygen species, including superoxide [28], where superoxide may contribute to aortic dissection and aneurysm progression. Elevated levels of superoxide in the aortic wall induce apoptosis of smooth muscle cells [32], [33], decreases elastin mRNA and protein expression [32], and increases fragmentation of the elastic lamellae [33]. We were therefore interested to see whether changes in superoxide production might accompany the protective effects of the high n-3 PUFA diet against neutrophil infiltration. The findings revealed a significant reduction in both Nox2 expression and amount of superoxide per adventitial inflammatory cell in both suprarenal and infrarenal segments. Since fewer macrophages and neutrophils infiltrated the adventitial region in ApoE^-/-^ mice receiving the high, compared to the low n-3 PUFA diet (significant for the infrarenal aortic segment), the findings suggest a substantially reduced burden of inflammatory cell-derived oxidative stress in the vessel wall. In addition, to the increased number of macrophages and neutrophils infiltrating the dissected, compared to the non-dissected aortas, Nox2 expression and superoxide production per cell was lower in mice receiving the high, compared to the low n-3 PUFA diet. Consistent with these findings, dissected aortas sometimes contained regions of media that was fibrotic and devoid of elastic lamellae (Figure S1). In mice that were infused for a longer duration with angiotensin II (2 week infusion), we observed a trend for delay to time of aortic rupture, although no significant difference in mortality attributed to rupture of the abdominal aorta. Studies are required to determine whether a high n-3 PUFA diet might also attenuate progression of disease when dietary supplementation commences after the establishment of the inflammatory response.

In conclusion, our study showed a beneficial effect of a high versus low n-3 PUFA diet in ApoE^-/-^ mice that were infused for a short (2 day) duration with angiotensin II. We report greater incorporation of n-3 PUFAs into membrane phospholipids in mice receiving the high, compared to the low n-3 PUFA diet. The combination of pro-inflammatory stimulus with a high n-3 PUFA diet increased plasma levels of the pro-resolving mediator, resolvin D1. The high n-3 PUFA diet also attenuated the infiltration of macrophages and neutrophils into the adventitial region of the vessel, and protected the mice against an oxidative stress response involving superoxide production by the infiltrating macrophages and neutrophils.

## Supporting Information

Figure S1 **A dissected abdominal aorta in an apolipoprotein E-deficient (ApoE^-/-^) mouse fed for 8 weeks on a low n-3 PUFA diet and infused with angiotensin II for two days.** The aorta contains a large intramural hematoma. Some regions of aorta were devoid of elastin fibres (arrows; van Gieson staining). Scale bar, 50 µm.
(TIF)

Table S1 **Plasma triglyceride concentration in C57bl/6 and apolipoprotein E-deficient (ApoE^-/-^) mice fed for 8 weeks on a low or high n-3 PUFA diet and infused with angiotensin II for two days.**
(DOCX)
