# Supplementary figures and images for: N-3 PUFAs Protect against Aortic Inflammation and Oxidative Stress in Angiotensin II-Infused Apolipoprotein E-/- Mice
Source: PLoS One. 2014 Nov 14;9(11):e112816. doi: 10.1371/journal.pone.0112816 (PMC4232505; doi:10.1371/journal.pone.0112816)

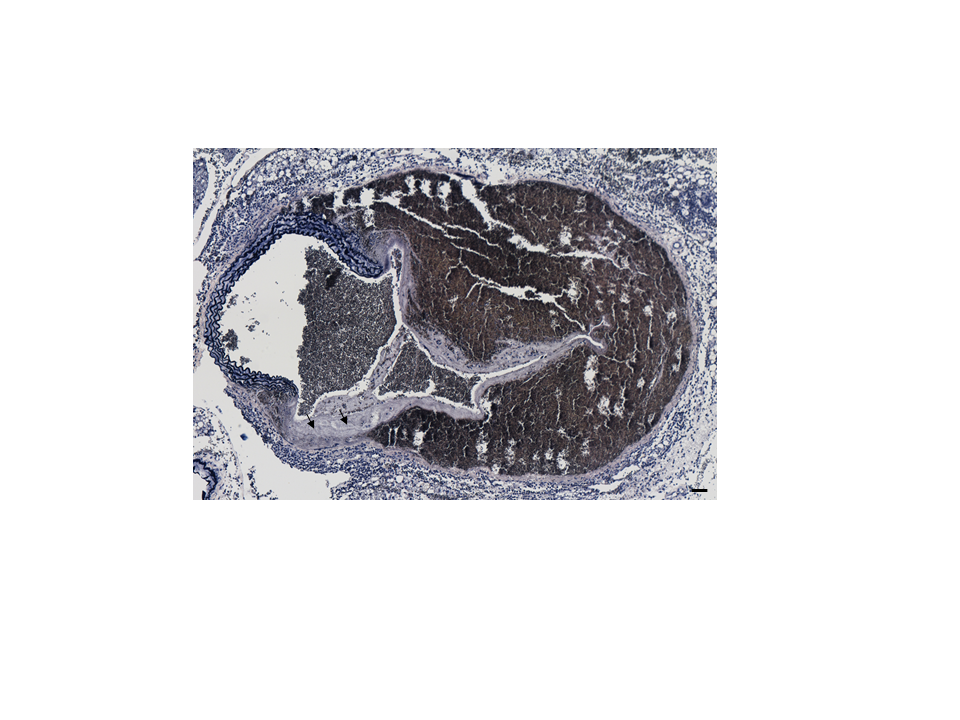

Supplement: Figure S1 — A dissected abdominal aorta in an apolipoprotein E-deficient (ApoE-/-) mouse fed for 8 weeks on a low n-3 PUFA diet and infused with angiotensin II for two days. The aorta contains a large intramural hematoma. Some regions of aorta were devoid of elastin fibres (arrows; van Gieson staining). Scale bar, 50 µm. (TIF) [file pone.0112816.s001.tif]
