# Supplementary material for: N-3 PUFAs Protect against Aortic Inflammation and Oxidative Stress in Angiotensin II-Infused Apolipoprotein E-/- Mice
Source: PLoS One. 2014 Nov 14;9(11):e112816. doi: 10.1371/journal.pone.0112816 (PMC4232505; doi:10.1371/journal.pone.0112816)
Supplement: Table S1 — Plasma triglyceride concentration in C57bl/6 and apolipoprotein E-deficient (ApoE-/-) mice fed for 8 weeks on a low or high n-3 PUFA diet and infused with angiotensin II for two days. (DOCX) [file pone.0112816.s002.docx]

|  | C57 Saline | | ApoE^-/-^ Angiotensin II | |
| --- | --- | --- | --- | --- |
| Lipid | Low diet | High diet | Low diet | High diet |
|  |  |  |  |  |
| Triglycerides (mmol/L) | 0.63±0.06 | 0.73±0.11 | 1.39±0.44 | 1.12±0.33 |

**Table S1** Total plasma triglyceride concentrations in C57 and apolipoprotein E^-/-^ mice receiving a low or high n-3 PUFA diet for 8 weeks. Data is expressed as mean±SEM.
